# Occupational Exposure to Pesticides as a Risk Factor for Sleep Disorders

**DOI:** 10.3390/ijerph20043149

**Published:** 2023-02-10

**Authors:** Ruirui Zheng, Jessica García-González, Raúl Romero-del Rey, Antonia López-Villén, Rafael García-Alvarez, Rosario Fadul-Calderon, Mar Requena-Mullor, Raquel Alarcón-Rodríguez

**Affiliations:** 1 Department of Nursing, Physiotherapy, and Medicine, University of Almería, 04120 Almería, Spain; 2 Torrecárdenas Hospital, 04009 Almería, Spain; 3 Faculty of Health Sciences, Human Sexuality Institute, Autonomous University of Santo Domingo, Santo Domingo 10103, Dominican Republic

**Keywords:** farmers, occupational exposure, pesticides, sleep disorder

## Abstract

Inadequate sleep has been linked to a variety of impairments in bodily functions, including endocrine, metabolic, higher cortical function, and neurological disorders. For this reason, the aim of this study was to analyze the link between occupational pesticide exposure and sleep health among farmers in Almeria. A cross-sectional study was conducted among a population living on the coast of Almeria (southeastern Spain), where about 33,321 hectares of land are used for intensive agriculture in plastic greenhouses. A total of 380 individuals participated in the study: 189 greenhouse workers and 191 control subjects. The participants were contacted during their annual scheduled occupational health survey. Data on sleep disturbances were collected using the Spanish version of the Oviedo Sleep Questionnaire. Agricultural workers were found to be at a significantly higher risk of insomnia, especially among those who did not wear protective gloves (OR = 3.12; 95% C.I. = 1.93–3.85; *p* = 0.04) or masks (OR = 2.43; 95% C.I. = 1.19–4.96; *p* = 0.01). The highest risk of insomnia related to pesticide applicators was observed in those who did not wear a mask (OR = 4.19; 95% C.I. = 1.30–13.50; *p* = 0.01) or goggles (OR = 4.61; 95% C.I. = 1.38–10.40; *p* = 0.01). This study supports previous findings indicating an increased risk of sleep disorder in agricultural workers exposed to pesticides at work.

## 1. Introduction

All living things require sleep, and humans spend one third of their lives doing so [1]. Inadequate sleep has been linked to a variety of impairments in bodily functions, including endocrine [2], metabolic [3], higher cortical function [4], and neurological disorders. Sleep disorders can cause difficulty sleeping, an excessive sense of well-being, or abnormal movements during sleep. According to a database of Western populations, the prevalence of various sleep disorders in the general population ranges from 0.047% to 50.5% [5]. According to the Spanish Society of Neurology (SEN), more than 4 million Spanish adults were suffering from chronic insomnia in 2015. By 2021, it is estimated that between 20 and 48% of the Spanish adult population will have difficulty initiating or maintaining sleep, and that at least 10% of these cases will be due to a chronic and severe sleep disorder, a figure that could be even higher due to the high number of undiagnosed cases. Even though many sleep disorders are treatable or preventable, less than one-third of patients seek professional assistance [6].

There are multiple risk factors associated with sleep disorders, including lifestyle factors such as a lack of exercise and poor diet [7], psychological factors such as stress and depression [8], and environmental factors such as noise and light [9]. Furthermore, chemical exposure can significantly impact sleep quality through various mechanisms and molecular pathways [2]. Over the last 15 years, various types of pesticides such as organochlorines, organophosphates and inorganic pesticides have been used in intensive agriculture [10]. Currently, some of these compounds, such as organochlorines, are banned from use. Carbamates, pyrethroids, and neonicotinoids are the most widely used pesticides for pest control. Pesticides have been widely used to control and prevent pests in homes and agriculture for the past 50 years [11]. Spain was the country in the European Union (EU) that sold the most pesticides in 2020, reaching nearly 76,024 tons of product, of which 10,073 tons (13.2%) were used in Almeria [12] (South Spain) in areas of intensive farming, both in greenhouses (particularly near the coastline of Almeria) and in traditional open-air agriculture (in inland areas). Farming practices that heavily rely on pesticides create a hazardous environment for human health, increasing the likelihood of developing diseases [13,14].

In previous studies, several researchers found that environmental exposure to pesticides was associated with a greater prevalence rate and a higher risk of having neurological disorders [15,16,17]. However, little is known about the effects of pesticide exposure on sleep. Occupational pesticide exposure was linked to sleep problems in a study of farmworkers with sleep behavior disorder in Uganda [11], China [18] and the United States [19,20], and in a multi-center study involving ten countries [21]. However, these studies did not discriminate between the wide range of pesticide groups or active ingredients used. Other reports indicate associations between occupational exposure to Ops [22,23], high incidence of dream-enacting behaviors [21,24] and sleep apnea [25]. To our knowledge, there are currently no studies in Spain that analyze the prevalence of sleep disorders in people exposed to pesticides, as is the case with farmers. The current study aimed to analyze the link between occupational pesticide exposure and sleep health among farmers in Almeria. The study’s findings can be used to create strategies for addressing mental health issues and promoting mental health and quality of life.

## 2. Materials and Methods

### 2.1. Design

A cross-sectional study was conducted on a population living on the coast of Almería (southeastern Spain), where about 33,321 hectares [26] of land are used for intensive agriculture in plastic greenhouses.

### 2.2. Study Population and Data Collection

A total of 380 individuals aged between 18 to 65 years were included in the study that took place from June 2021 to July 2022. The participants were contacted during their annual scheduled occupational health survey. The exposed group consisted of 189 greenhouse workers undertaking activities such as pruning, weeding, thinning, and pesticide application in the greenhouses (with an average area of 1 ha) for 40 h per week for at least 2 consecutive years. The pesticides most used on greenhouse crops during the study period (tomato, cucumber, melon, watermelon, and zucchini) are listed in Table 1. The inclusion criteria for the exposed group were: to be a greenhouse farmer over 18 years of age, to be male, to have lived at their current address for at least 5 years, and to have been engaged in greenhouse farming for more than 2 years. The control group consisted of 191 healthy individuals with no previous or current occupational exposure to pesticides. The control subjects lived in the same geographic area as the greenhouse workers to minimize any differences in background exposure. The inclusion criteria for the control group participants were: to be a worker over 18 years of age, to be male, and to not be undertaking or have undertaken agricultural activities.

Data on sleep disturbances were collected using the Spanish version of the Oviedo Sleep Questionnaire [27]. The questionnaire is a brief semi-structured interview used as a diagnostic aid for insomnia and hypersomnia type sleep disorders. The questionnaire consists of 15 items, 13 of which are grouped into three subscales: subjective sleep satisfaction (one item), insomnia (nine items) and hypersomnia (three items). The remaining two items provide information on the use of sleep aids or the presence of adverse phenomena during sleep. All items are scored from one to five points, except the first item, which is scored from one to seven points. The total score ranges from 9 to 45 points, with a higher score indicating greater severity. The psychometric properties obtained confirm this questionnaire is a valid instrument for patients with sleep disorders (Cronbach’s alpha coefficient: 0.77).

The questionnaires were completed and information on sociodemographic variables (age, level of education, smoking, body mass index) was gathered via a face-to-face interview conducted by a trained interviewer and a psychiatrist. In addition, the farmers were interviewed to gather information on the agricultural tasks performed, years spent working in agriculture, application of pesticides and use of personal protective equipment (PPE, e.g., gloves, goggles, face mask, protective clothing).

### 2.3. Ethical Considerations

The research study was approved by the Ethics and Research Committee of the University of Almeria (EFM 96/2021). Participation in the study was voluntary. The participants signed an informed consent form after being informed of the study’s objectives. All of the procedures were performed following the ethical standards of the Helsinki Declaration. A personal code was generated for each participant to ensure data confidentiality.

### 2.4. Data Analysis

A database with the study variables was created with IBM SPSS statistical software (SPSS 26.0 for Windows, IBM, Armonk, NY, USA). Descriptive analysis was performed using means and standard deviations for continuous variables, whereas absolute and relative frequency distributions were calculated for dichotomic and categorical variables. The Kolmogorov–Smirnov test was used to assess whether the continuous variables (age, insomnia level, hypersomnia level and years working in agriculture) were normally distributed. As these continuous data were non-normally distributed, the non-parametric Mann–Whitney U test was used for further comparisons. Chi-square tests were performed to evaluate the distribution of dichotomic and categorical variables for the outcome of the study. The risk of insomnia was calculated for farmers and pesticide applicators using odds ratios (OR) and the 95% confidence interval (CI).

Multiple binary logistic regression analysis was used to assess the risk of insomnia among the farmers and pesticide applicators. Models were adjusted for variables that showed statistically significant differences in the bivariate analysis. The level of statistical significance was set at a *p*-value < 0.05.

## 3. Results

A total of 380 male workers participated in the study: 189 greenhouse workers (farmers) and 191 control subjects (non-agricultural). The two study groups did not differ with respect to possible confounding factors, such as age, smoking status and BMI (Table 2). In contrast, statistically significant differences were observed at the education level. Therefore, this variable was included in the multivariate analysis as a potential confounder.

Notably, 83.1% of the greenhouse workers and 56.03% of the control subjects had sleep problems. The farmers had a higher score than the non-agricultural workers for insomnia (30.16 ± 3.50 and 22.70 ± 3.96, respectively) and hypersomnia (9.56 ± 2.11 and 8.24 ± 1.77, respectively). According to the Oviedo Sleep Questionnaire, 77.8% of greenhouse workers and 15.2% of non-agricultural workers had insomnia. All these results were statistically significant.

Table 3 shows the risk of insomnia for farmers and sprayers separately, expressed as OR, and adjusted for use of no PPE during their working day vs. use of any PPE, years working in agriculture, and devices used for pesticide application. A significantly increased risk of insomnia was found in farmers who did not use gloves and masks while farming, compared with their respective reference groups (no use of mask: OR = 2.13, *p* < 0.05; no use of gloves: OR = 2.06, *p* < 0.05).

The highest risk of insomnia was found among sprayers who did not use goggles (OR of 3.22; *p* < 0.05) and who did not wear a face mask during the application of pesticides (OR 3.12; *p* < 0.05). Farmers with insomnia had been working in agriculture for almost the same number of years as those without insomnia. Conversely, in the group of sprayers, those with insomnia had been working in agriculture for less time (years) than those without insomnia, with the differences not being statistically significant in either case (Table 3).

Table 4 shows the results of the multiple binary logistic regression analysis of possible factors that may be associated with insomnia in farmers and pesticide applicators (sprayers). The model for farmers was adjusted for the following independent variables: age, education level, years worked in agriculture, and use of gloves and face mask as PPE. The highest risk of insomnia was observed among farmers who did not use gloves on workdays (OR = 3.12, *p* < 0.05) and for those who did not wear a mask (OR = 2.43, *p* < 0.01).

For pesticide applicators, the model was adjusted for age, type of agriculture, education level, use of mask and use of goggles, as these showed a significant association in the bivariate analyses (Table 4). The highest risk of insomnia was found for sprayers who did not use goggles while spraying (OR = 4.61, *p* < 0.01) and for those who did not wear a mask (OR = 4.19, *p* < 0.01).

## 4. Discussion

The present study sought to analyze the link between occupational pesticide exposure and sleep health among farmers in Almeria. To our knowledge, there are currently no studies in Spain that analyze the prevalence of sleep disorders in people exposed to pesticides, such as farmers.

Most pesticides have detrimental effects on human health, which occur due to their impact on neurological functions, such as inhibition of acetylcholinesterase [28,29], leading to the related nerves being easily activated. Short sleep duration indicates stress, nervous disruption, or highly reactive nerve cells. In addition, pesticide exposure could reduce sleep time and sleep duration. These findings are consistent with the study by Yi-chun [30], who indicated that long-term exposure to pesticides leads to disruption of sleep and wake function, which may result in poor sleep quality. Another study conducted by Li et al. [18] also reported associations between medium and high cumulative exposure intensity and sleep disturbances (short sleep duration, poorer perceived sleep quality and insomnia) among greenhouse farmers.

In a recently published study, researchers reported an increased risk of sleep problems in addition to insufficient sleep among farmers exposed to pesticides [11]. These results coincide with ours, as farmers in our study presented greater sleep problems, insomnia and hypersomnia with statistically significant differences compared to non-farmers. Other authors have found an increased risk of snoring [11] and sleep apnea after low-level exposure to carbamates among farmers [25]. Sleep disturbances are an underlying indicator of psychological symptoms; adults who experience sleep disturbances are up to 3.6 times more likely to have depressive symptoms [31,32].

Several factors influence the occurrence of adverse health effects following occupational exposure to pesticides, such as mixing and spraying pesticides; wind/drift of agricultural pesticides; lack of, or improper use of PPE, knowledge, or perceptions; wearing contaminated clothing; hot weather; eating; drinking; smoking during or after pesticide use; and personal hygiene (hand washing or showering) [33]. There is evidence confirming that farmers who follow pesticide management and PPE recommendations have a significantly lower risk of having high-levels of pesticide metabolites in their urine [34].

Existing evidence confirms that the use of PPE on pesticide applicators and by farmers significantly reduces exposure levels and health problems [33,34]. PPE is an important modifier of occupational exposure to pesticides and is essential to reducing the risk of developing pesticide-related symptoms and illnesses [35]. Although it is in a transitional phase, crop protection is still largely achieved through the application of chemicals [36]. Biological crop control methods have contributed to a significant decline in the use of pesticides and other chemical agents. However, they are still a resource used for pest control, meaning that there is a need to protect workers, hence the importance of using PPE [35]. Applicators and farmers have the highest exposure to these compounds, with skin and inhalation being the most common means of exposure [37]. Several studies indicate that the PPE most used by pesticide handlers are long-sleeved shirts and pants, goggles and caps [38,39]. In our study, 100% of sprayers wore protective clothing; however, most farmers and sprayers did not wear gloves, goggles and masks as PPE (Table 3). A previous study also reported that only 17% of farmers were well protected based on FAO recommendations [34]. The reasons for non-compliance with PPE use by pesticide handlers include factors such as discomfort during PPE use [40], climatic conditions [41], and the low availability and cost associated with protective equipment [42]. Farmers’ use of PPE depends on their perception of the health risks of pesticides [43].

Our study found that farmers who did not wear gloves while handling pesticides had three times the risk (OR = 3.12) of insomnia compared to those who did wear gloves. In relation to the use of masks, farmers who did not use them had a significantly higher risk (OR = 2.43) of insomnia. Pesticide applicators who did not wear masks and goggles had four times the risk (OR = 4.19; OR = 4.61, respectively) of having insomnia compared to pesticide applicators who did, demonstrated by statistically significant differences. Therefore, the use of PPE and adherence to safe practices during the handling of pesticides in agricultural activities can reduce the total exposure to pesticides [37] and thus avoid the negative effect of pesticides on health and the occurrence of some disorders, such as sleep disorders.

A lack of information on specific pesticide exposure may limit our findings. There could be a memory recall bias related to pesticide use and personal information, which could impact the accuracy of the results. In addition, sleep problems were assessed at a specific point in time, so this measure may not reflect actual changes in sleep status. Therefore, future studies should follow a cohort study design and measure sleep disorders using activity meters or polysomnography to determine sleep quality.

## 5. Conclusions

Our findings suggest an increased risk of sleep disorders in farmers occupationally exposed to pesticides. The risk of insomnia was higher for farmers who did not wear gloves or masks as a protective measure to minimize exposure when carrying out their work tasks. As for pesticide applicators, the highest risk of insomnia was observed in those who did not wear a mask or goggles.

## Figures and Tables

**Table 1:** Pesticides most often used in greenhouses from the study area over the last 15 years.

Active Substance	Action	Chemical Class	Crops Treated	Year of Ban
Carbendazim	Fungicide	Benzimidazole	Cucumber	2014
Chlorpyrifos-methyl	Insecticide	Organophosphate	Tomato/pepper/cucumber/melon/watermelon	2020
Endosulfan	Insecticide	Organochlorinated (cyclodiene)	Tomato/pepper	2007
Famoxadone	Fungicide	Oxazolidinedione	Tomato/cucumber/melon	2022
Flutriafol	Fungicide	Triazole	Tomato/pepper	2021
Folpet	Fungicide	Dicarboximide	Tomato/cucumber	2023 ^b^
Formetanate	Insecticide	*N*-methylcarbamate	Aubergine/tomato/pepper/zucchini	2023 ^b^
Imidacloprid ^a^	Insecticide	Neonicotinoid	Aubergine/tomato/zucchini	2020
Mancozeb	Fungicide	Dithiocarbamate	Tomato/pepper/melon/watermelon	2021
Methiocarb	Insecticide	*N*-methylcarbamate	Tomato/pepper/cucumber	2019
Methomyl	Insecticide	*N*-methylcarbamate	Aubergine/tomato/pepper/melon/watermelon	2019
Naled	Insecticide	Organophosphate	Zucchini	2012
Propineb	Fungicide	Dithiocarbamate	Tomato/pepper/cucumber	2019
Pymetrozine	Insecticide	Pyridine azomethine	Aubergine/tomato/pepper/zucchini/cucumber	2015
Pyrimethanil	Fungicide	Pyrimidine	Aubergine/tomato/pepper/cucumber	2023 ^b^
Thiophanate-methyl	Fungicide	Benzimidazole	Aubergine/tomato/melon/watermelon	2020

^a^ Allowed in the EU only if used as an insecticide in permanent greenhouses. ^b^ Expiration of approval in the European Union (EU).

**Table 2:** Comparison of sociodemographic data, smoking habits and sleep disorders between the two study groups, ‘agricultural occupation’ and ‘non-agricultural’.

Characteristic		Agricultural Occupation (n = 189)	Non-Agricultural (n = 191)	*p* Value
Age (years) ^†^		39.04 ± 5.33	39.50 ± 8.46	0.520 *
Education level (%)	No studies	55 (29.1)	26 (13.6)	0.001 **
	Low	72 (38.1)	69 (36.1)	
	Medium	50 (26.5)	73 (38.2)	
	High	12 (6.3)	23 (12.1)	
Tobacco (%)	Yes	93 (49.2)	89 (46.6)	0.155 **
	No	72 (38.1)	87 (45.5)	
	Former smoker	24 (12.7)	15 (7.9)	
BMI (%)	Normal (18.5–24.9)	127 (67.2)	113 (59.2)	0.060 **
	Low (<18.5)	23 (12.2)	30 (15.7)	
	High (≥25.0)	39 (20.6)	48 (25.1)	
Sleep problem (%)	Yes	157 (83.1)	107 (56.03)	0.001 **
	No	32 (16.9)	84 (43.97)	
Insomnia level ^†^ (Median: 27)		30.16 ± 3.50	22.70 ± 3.96	0.001 *
Hypersomnia level ^†^ (Median: 9)		9.56 ± 2.11	8.24 ± 1.77	0.001 *
Insomnia (%)	Yes	147 (77.8)	29 (15.2)	0.001 **
	No	42 (22.2)	162 (84.8)	

^†^ Data expressed as mean ± standard deviation; BMI: Body Mass Index. *p* value obtained using * Mann–Whitney *U* test for continuous variables or ** Chi-squared test for categorical variables.

**Table 3:** Risk of insomnia among farmers and sprayers in relation to use of PPE and devices used for pesticide application.

Characteristic		Farmers				Sprayers (Pesticide Applicators)			
		Insomnia (n = 147)	Non-Insomnia (n = 42)	OR	*p* Value	Insomnia (n = 72)	Non-Insomnia (n = 16)	OR	*p* Value
PPE (use of gloves)	No	102 (69.4%)	22 (52.4%)	2.06	0.041 **	45 (62.5%)	9 (56.3%)	1.29	0.857 **
	Yes	45 (30.6%)	20 (47.6%)			27 (37.5%)	7 (43.8%)		
PPE (use of goggles)	No	115 (78.2%)	28 (66.7%)	1.79	0.123 **	58 (80.6%)	9 (56.3%)	3.22	0.039 **
	Yes	32 (21.8%)	14 (33.3%)			14 (19.4%)	7 (43.8%)		
PPE (use of mask)	No	106 (72.1%)	23 (54.8%)	2.13	0.033 **	51 (70.8%)	7 (43.8%)	3.12	0.044 **
	Yes	41 (27.9%)	19 (45.2%)			21 (29.2%)	9 (56.3%)		
PPE (use of protective clothing)	No	79 (53.7%)	16 (38.1%)	1.88	0.074 **	-	-	-	-
	Yes	68 (46.3%)	26 (61.9%)			72 (100%)	16 (100%)		
Years working in agriculture		14.37 ± 7.14	14.83 ± 6.26	-	0.706 *	10.86 ± 6.48	11.19 ± 6.40	-	0.856 *
Devices used for pesticide application	Spray gun	-	-	-	-	27 (37.5%)	7 (43.8%)	0.77	0.642 **
	Cannon spraying machine	-	-			45 (62.5%)	9 (56.2%)		

*p* value obtained using * Mann–Whitney *U* test for continuous variables or ** Chi-squared test for categorical variables.

**Table 4:** Stepwise multiple binary logistic regression analysis of the risk of insomnia in farmers and applicators, adjusted for potential risk factors.

	Parameters	OR	95% C.I.	*p*-Value
Farmers ^a^	Use of gloves (no)	3.12	1.93–3.85	0.04
	Use of a face mask (no)	2.43	1.19–4.96	0.01
Sprayers (pesticide applicators) ^b^	Use of goggles (no)	4.61	1.38–10.40	0.01
	Use of a face mask (no)	4.19	1.30–13.50	0.01

^a^ The regression model was adjusted for age, education level, use of gloves (0: yes; 1: no), use of a mask (0: yes, 1: no). ^b^ The regression model was adjusted for age, education level, use of goggles (0: yes; 1: no) use of a mask (0: yes, 1: no).

## Data Availability

Data of this study are stored in an SPSS software Project.
